# K-wire versus screws in the fixation of lateral condyle fracture of humerus in pediatrics: a systematic review and meta-analysis

**DOI:** 10.1186/s12891-023-06780-5

**Published:** 2023-08-12

**Authors:** Yoon Joo Cho, Se Hyun Kang, Mu Hyun Kang

**Affiliations:** Department of Orthopaedic Surgery, St. Vincent’s Hospital, College of Medicine, The Catholic University of Korea, 222, Banpo-Daero, Seocho-Gu, 06591 Seoul, Korea

**Keywords:** Lateral condyle fracture of the humerus, K-wire fixation, Screw fixation, Meta-analysis

## Abstract

**Background:**

Lateral condyle fracture of the humerus is the second most common elbow fracture in children. Non-displaced lateral condyle fractures can often be managed with cast and conservative care, while reduction and fixation are often used to treat displaced lateral condyle fractures. Traditionally, K-wire fixation has been used for displaced lateral condyle fractures, but recently fixation using screws has been advocated in some studies. Therefore, we performed a meta-analysis to determine the difference in outcomes and complications between the two different fixation methods for the treatment of displaced lateral condyle fractures of the humerus in pediatric patients.

**Methods:**

Preferred Reporting Items for Systematic Reviews and Meta-Analyses (PRISMA) guidelines were used for this review. PubMed, Embase, and Cochrane Library were used for study selection. Studies comparing K-wires and screw fixation in displaced lateral condyle fractures in pediatric patients were included. Clinical outcomes using the Hardacre criteria, infection, limitation of range of motion of the elbow, lateral condyle overgrowth, delayed union, nonunion, and avascular necrosis were compared. Data were analyzed using the meta package in R version 4.2.2, and random-effects or fixed-effects models were used according to heterogeneity.

**Results:**

One randomized controlled study and three retrospective cohort studies were included, with a total of 240 patients (K-wire:118, screw:122). The clinical outcome using the Hardacre criteria was not different between the groups (*P* = 0.54), but the risk of infection (risk ratio [RR] = 5.52, 95% CI: 1.42–21.48, *P* = 0.01) and limitation of range of motion (RR = 3.75, 95% CI: 1.54–9.18, *P* < 0.01) were significantly higher in the K-wire fixation group than in screw fixation group.

**Conclusions:**

The use of screws for fixation after reduction in the treatment of lateral condyle fracture of the humerus in children decreases the risk of superficial infection and elbow stiffness compared with the use of K-wire. Although removal of the implant under general anesthesia is necessary, screw fixation can be considered in displaced lateral condyle fractures of the humerus in children.

**Trial registration:**

PROSPERO (CRD42023415643).

**Supplementary Information:**

The online version contains supplementary material available at 10.1186/s12891-023-06780-5.

## Background

In pediatrics, lateral condyle fracture is the second most common fracture of the elbow, and encompasses approximately 12% to 20% of all distal humerus fractures in children [1]. Nondisplaced fractures are usually treated with a long arm cast [2–7], but fractures with initial displacement tend to displace further and have a high incidence of nonunion, necessitating reduction and fixation [8]. Traditionally, in these fractures, open reduction and internal fixation were preferred; however, in recent studies, closed reduction and percutaneous pinning has been reported to produce similar outcomes to open reduction and fixation [9–13]. However, studies showed different methods of fixation: K-wires were traditionally used, but fixation using screws also showed tolerable results [14–16]. There is a systematic review concluding that there is no difference between the outcomes between the K-wire fixation and screw fixation [17]. However, it was only qualitatively compared, and currently, there is no meta-analysis which synthesizing the results with quantitative analysis comparing K-wire fixation with screw fixation for displaced lateral condyle fractures of the humerus in pediatrics. Therefore, we designed a meta-analysis to determine the outcome of two different fixation methods for the treatment of displaced lateral condyle fractures of the humerus in pediatric patients to provide evidence for deciding the fixation method.

## Methods

The protocol of this study was registered in PROSPERO (ID: CRD42023415643). We followed the Preferred Reporting Items for Systematic Reviews and Meta-Analyses (PRISMA) guidelines for reporting this systematic review and meta-analysis.

### Search Strategy

The PubMed, Embase, and Cochrane Library databases were searched for articles published before April 10, 2023, that combined the terms “lateral condyle fracture of humerus” or “lateral condylar fractures of humerus” or “lateral condyle fracture of humerus” or “lateral condyle fractures of humerus” and “pediatric” or “children.”

### PICO (Population, intervention, comparison, and outcome)

PICO was defined as following; P: Displaced lateral condyle fracture in children under the age of 16, I: Screw fixation, C: K-wire fixation, O: Postoperative clinical outcome and complications including infection, delayed union, nonunion, lateral overgrowth, limitation of range of motion, and avascular necrosis.

### Study selection and data extraction

Two reviewers performed a literature search for studies comparing K-wire fixation and screw fixation in lateral condyle fractures in pediatric patients independently. Searches were conducted according to the Preferred Reporting Items for Systematic Reviews and Meta-Analyses (PRISMA) guidelines. Disagreements were resolved with the third researcher through discussion.

The inclusion criteria were articles with the preoperative diagnosis of displaced lateral condyle fracture in children under the age of 16 and including clinical outcomes of the K-wire and screw fixation groups. Studies that included combined screw and pin fixation were excluded. Only English language articles were included. Case reports, case series, reviews, systematic reviews, editorial letters, and articles without full text were excluded.

This study aimed to assess the primary clinical outcome with Hardacre criteria [18] and complications including infection, delayed union, nonunion, lateral overgrowth, limitation of range of motion, and avascular necrosis. Each outcome was defined if the studies indicated it as infection, delayed union, nonunion, lateral overgrowth, limitation of range of motion, and avascular necrosis, and specific definition of outcomes which were mentioned in each study are shown in supplement table II [19–22].

### Bias assessment

The risk of bias was assessed using the revised Cochrane risk of bias tool for randomized studies (ROB2) [23] and the Methodological Index for Non-Randomized Studies (MINORS) score for non-randomized studies [24]. Two independent reviewers assessed bias, and disagreements were resolved with the third author through discussion. Publication bias was assessed for the primary clinical outcome using funnel plot and Egger’s test.

### Statistical analyses

Data were analyzed using the “meta” package in R (version 4.2.2; R Foundation for Statistical Computing, Vienna, Austria). For binary data, risk ratio (RR) was used for the effect size. Heterogeneity was determined using I^2^ for each model; I^2^ < 25% was considered low heterogeneity. For low heterogeneity, fixed-effects model was used; otherwise, a random-effects model was used. In analyzing the nonunion and avascular necrosis results, only one study in each outcome showed more than one event in the K-wire fixation group but zero event in the screw fixation group, so the relative risk was analyzed by imputing the zero events in the screw fixation group with 0.5. Statistical significance was set at *P* < 0.05.

## Results

### Search results

**Fig. 1:**
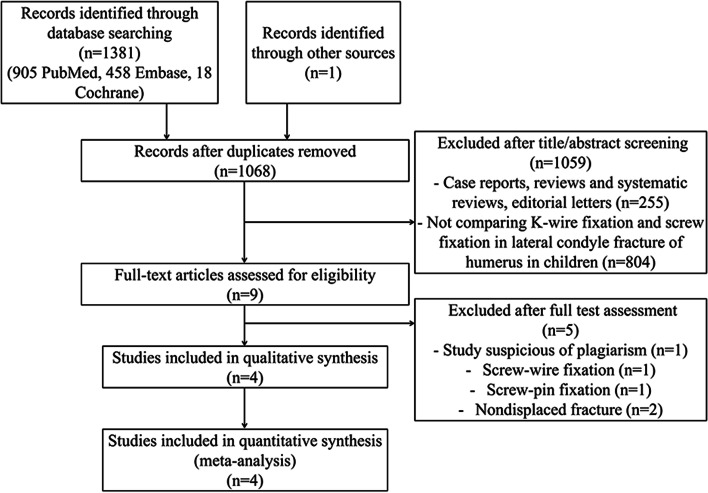
Preferred reporting items for Systematic Reviews and Meta-Analyses (PRISMA) diagram on study selection process

Initially, a total of 1381 studies were identified, and 1068 studies remained after duplicate removal. Nine studies were reviewed with full text, one study was removed due to suspect of plagiarism, two were removed because of screw-wire usage or combined screw-pin usage, and two were removed because they included nondisplaced fractures. Finally, four articles were selected for the meta-analysis. A flowchart of the study selection is shown in Fig. 1.

### Risk of bias assessment

The average MINORS score for non-randomized studies was 17.7 (range, 15 to 19) (Table 1), and the assessment of risk-of-bias for the randomized controlled trial is shown in Table 2. The funnel plot of primary clinical outcome with Hardacre criteria is shown in supplementary Figure I, and Egger’s test showed *P*-value of 0.34, meaning there was no publication bias.

**Table 1:** MINORS score for nonrandomized comparative studies

**Quality assessment for nonrandomized trials**	**Li** [20]	**Gilbert** [19]	**Stein** [22]
A clearly stated aim	2	2	2
Inclusion of consecutive patients	2	2	1
Prospective data collection	0	0	0
Endpoints appropriate to the aim of the study	2	2	2
Unbiased assessment of the study endpoint	2	2	2
A follow-up period appropriate to the aims of study	1	1	1
Less than 5% loss to follow up	2	2	0
Prospective calculation of the sample size	0	0	0
An adequate control group	2	2	2
Contemporary groups	2	2	2
Baseline equivalence of groups	2	2	1
Adequate statistical analyses	2	2	2

**Table 2:** Revised cochrane risk-of-bias assessment (ROB2)

	**Thapa** [21]
Randomization process	Low risk
Deviations from intended interventions	Low risk
Missing outcome data	High risk
Measurement of the outcome	Some concerns
Selection of the reported result	Low risk
Overall bias	High risk

### Study characteristics

One randomized controlled trial [21] and three retrospective cohort studies [19, 20, 22] were included, with a total of 240 patients (K-wire:118, screw:122). The demographic characteristics and details of the included studies are summarized in Table 3 and supplementary table II.

**Table 3:** Study characteristics

Studies	Country	Sample Size (K/S)	Mean Age	Gender (M/F)	Laterality (R/L)
**Li** [20]	China	30/32	6.9 years	42/20	37/25
**Gilbert** [19]	USA	43/41	5.6 years	59/25	Not reported
**Stein** [22]	USA	22/26	5.1/5.9 years	33/15	14/34
**Thapa** [21]	Nepal	23/23	6.6 years	34/12	24/22

### Outcomes of meta-analysis

#### Clinical outcome (Criteria of Hardacre)

**Fig. 2:**
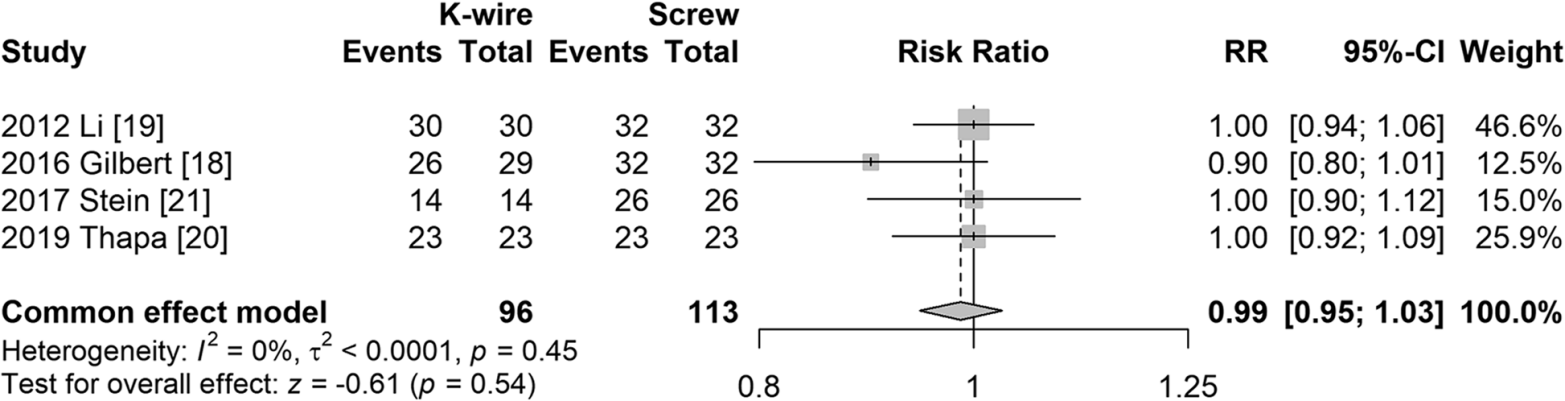
Forest plot of clinical outcome using Hardacre criteria

Four studies [19–22] reported clinical outcomes using the Hardacre criteria. Excellent and good results were considered satisfactory, while poor results were considered unsatisfactory. A fixed model was used, and no heterogeneity was found (I^2^ = 0%, *P* = 0.45). There was no difference between the clinical outcome using the Hardacre criteria between the groups (Fig. 2).

#### Infection

**Fig. 3:**
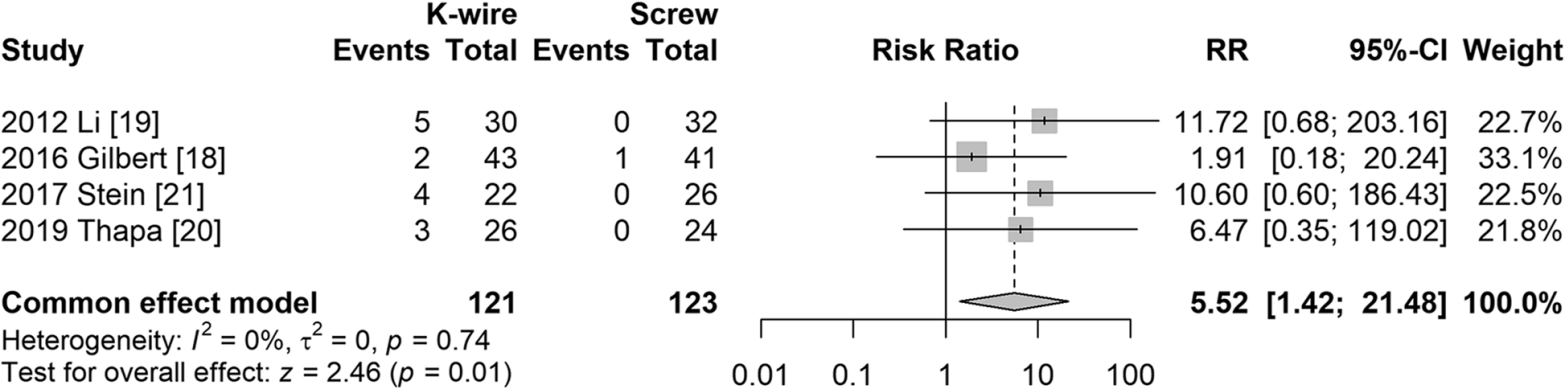
Forest plot of superficial infection

Four studies [19–22] reported infection. A fixed model was used owing to small heterogeneity (I^2^ = 0%, *P* = 0.74). The risk of infection was significantly higher in the K-wire fixation group (RR = 5.52, 95% CI: 1.42–21.48, *P* = 0.01) (Fig. 3).

#### Limitation of range of motion

**Fig. 4:**
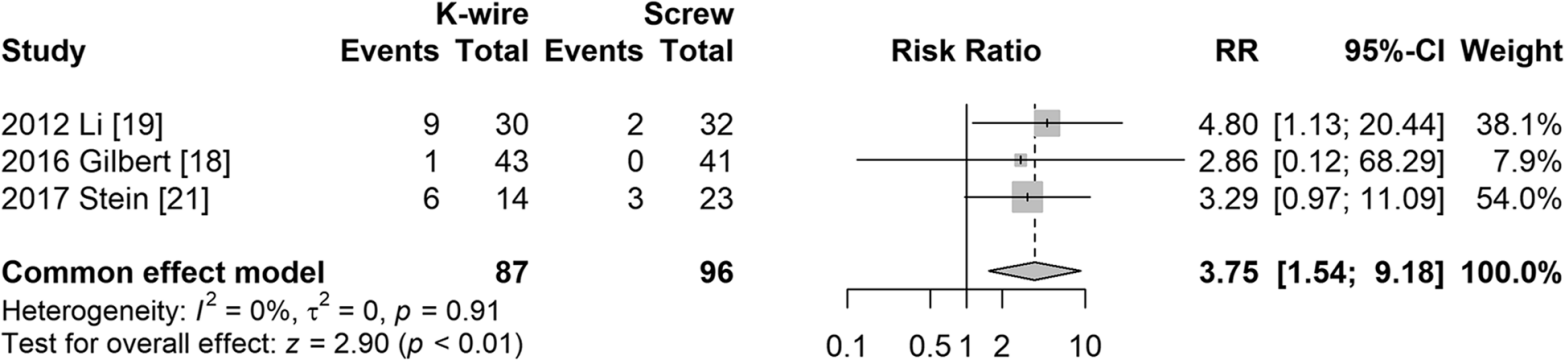
Forest plot of limitation of motion

Three studies [19, 20, 22] reported limitations in the range of motion. The fixed model was used due to no heterogeneity (I^2^ = 0%, *P* = 0.91). The risk of limitation of range of motion was significantly higher in the K-wire fixation group (RR = 3.75, 95% CI: 1.54–9.18, *P* < 0.01) (Fig. 4).

#### Lateral overgrowth

**Fig. 5:**
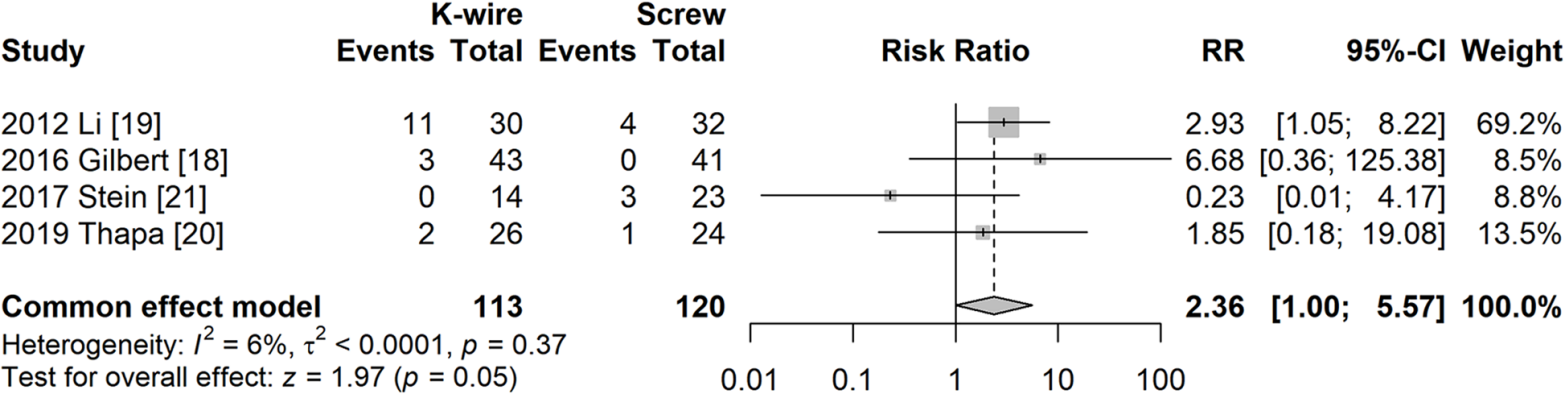
Forest plot of lateral overgrowth

Four studies [19–22] reported lateral overgrowth as an outcome. The fixed model was used due to low heterogeneity (I^2^ = 6%, *P* = 0.37). The risk of lateral overgrowth seemed to be higher in the K-wire fixation group, but this was not statistically significant (RR = 2.36, 95% CI: 1.00–5.57, *P* = 0.05) (Fig. 5).

#### Delayed union

**Fig. 6:**
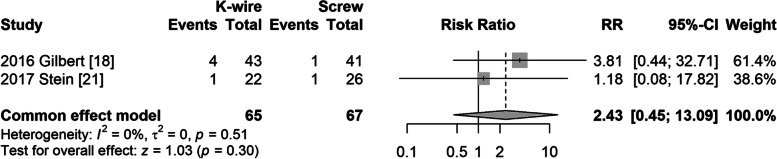
Forest plot of delayed union

Two studies [19, 22] reported delayed union as an outcome. The fixed model was used due to no heterogeneity (I^2^ = 0%, *P* = 0.51). The risk ratio between the groups was not significantly different (RR = 2.43, 95% CI: 0.45–13.09, *P* = 0.30) (Fig. 6).

#### Nonunion

**Fig. 7:**
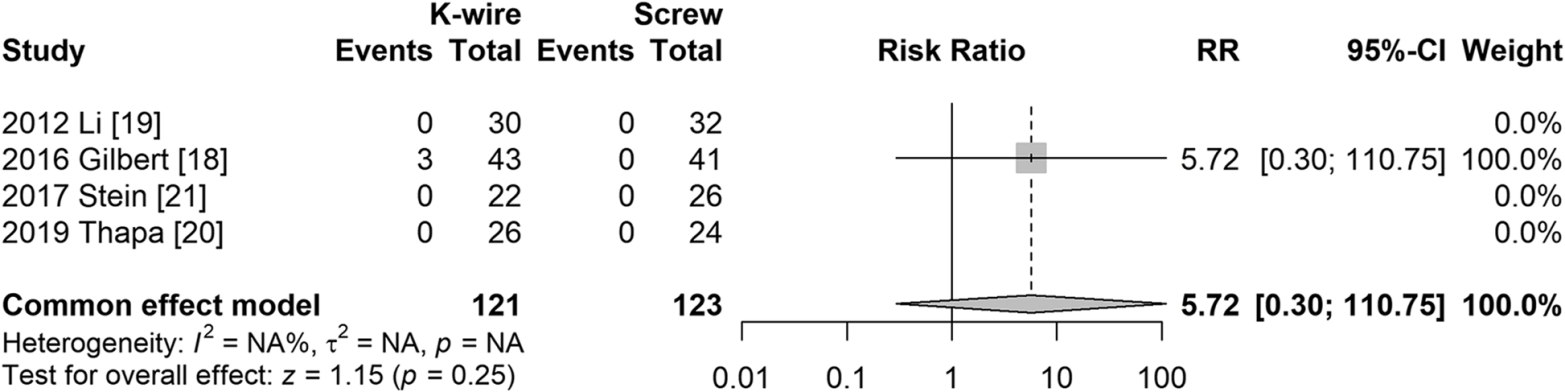
Forest plot of nonunion

Four studies [19–22] reported nonunion; however, only one study reported nonunion in the K-wire group. The risk ratio was not significantly different (RR = 5.72, 95% CI: 0.30–110.8, *P* = 0.25) (Fig. 7).

#### Avascular necrosis (Fishtail deformity and avascular necrosis of capitellum)

**Fig. 8:**
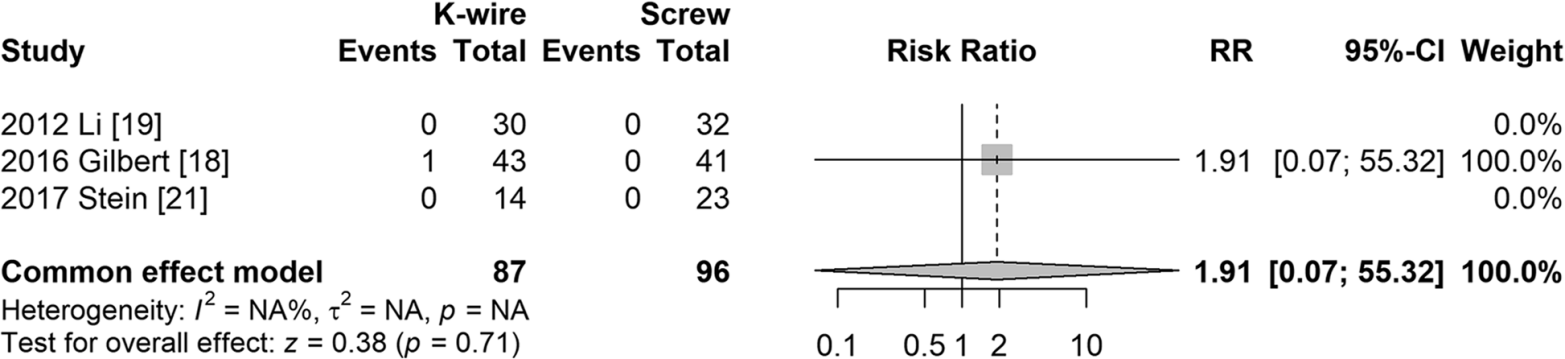
Forest plot of avascular necrosis

Three studies reported the outcome of avascular necrosis (AVN), but only one study reported nonunion in the K-wire group. The risk ratio was not significantly different (RR = 1.91, 95% CI: 0.07–55.3, *P* = 0.71) (Fig. 8).

## Discussion

The treatment of lateral condyle fractures of the humerus in children has been a topic of interest to researchers for decades. At present, minimal or nondisplaced lateral condyle fractures are usually treated with casting and short-term follow-up, due to the possibility of displacement [2–7]. Displaced lateral condyle fractures were treated surgically. Traditionally, open reduction and fixation were used, but some studies started advocating closed reduction and fixation, leading to a consensus of trying closed reduction first even though the displacement is more than 4 mm, and if the post-reduction displacement is more than 2 mm, open reduction, and fixation are used [9–13]. However, controversy still exists regarding the method of fixation.

Traditionally, K-wires have been used for the fixation of lateral condyle fractures of the humerus in children [9, 10, 25, 26]. Before screws were advocated as a fixation material, most studies did not focus on the fixation material. They were more oriented to open reduction versus closed reduction in displaced lateral condyle fractures or the outcomes and complications after lateral condyle fractures. However, fixation material beside K-wires began to emerge as a topic of lateral condyle fracture research due to the concern of pin infection, loss of fixation, and longer duration time of casting, resulting in a limited range of motion. Biodegradable materials [27–30], screw-wire [31], and screws [14–16, 32, 33] were studied as fixation materials. Screw fixation showed satisfactory outcomes, and the studies showed that the concern of screw-related growth plate complications was not true [14–16].

Comparative studies on K-wire fixation and screw fixation have been published [19–22, 34–38]. An abstract was reported as the first study to compare K-wire and screw fixation in lateral condyle fractures, and the results showed no difference between the groups [38]. However, two biomechanical studies showed significant mechanical advantages of screw fixation using a bone model, theoretically leading to improved healing, decreased casting duration, and faster return of range of motion [35, 37]. In clinical comparative studies, Li et al. [20] reported similar outcomes using the Hardacre criteria, but lower rates of infection, limitation of range of motion, and lateral condyle overgrowth in the screw fixation group than in the K-wire fixation group. They did not use casting or bracing period as an outcome, but uniformly applied above-elbow plaster splint for 5 to 6 weeks in the K-wire group and 1 to 2 weeks for the screw fixation group, resulting in a shorter duration of the brace, resulting in faster starting time in exercising range of motion, which might have resulted in the difference of limitation of range of motion in the two groups. Gilbert et al. [19] reported similar results. The screw fixation group had shorter time to union and days of casting, and lower overall complication rates than the K-wire fixation group. Stein et al. [22] also reported a shorter casting time, an earlier range of motion, and lower infection rates in the screw fixation group. They also emphasized that closed reduction can be accomplished more frequently with screw fixation than with K-wire fixation. Thapa et al. [21] reported a lower superficial infection rate and lateral condyle overgrowth in the screw fixation group. They did not use the cast fixation period as the outcome but treated the K-wire fixation group with a splint for 4 weeks after which the range of motion was started. The screw fixation group was treated for 2 weeks, which was much faster.

To our knowledge, this is the first meta-analysis to compare K-wire and screw fixation in displaced lateral condyle fractures of the humerus in children. As reported in previous studies, the risk of superficial infection and elbow stiffness was lower in the screw fixation group than in the K-wire fixation group. Patients who underwent screw fixation, the screw was deep inside the skin, which may explain the lower infection rate. Although we could not quantitatively synthesize the time to union or the time of cast due to limited reports of the outcome, as we discussed earlier, the screw fixation group usually had a shorter time of cast, leading to a faster start in the range of motion, which might have led to a lower risk of elbow stiffness. The risk of lateral condyle overgrowth was reported to be higher in the K-wire fixation group in some studies, and our meta-analysis also showed a higher risk ratio, but it was not statistically significant. The nonunion rate was not different between the groups. This implies that the K-wire is sufficiently strong for the fracture to be fixated in the reduction state if it is properly fixated.

Two studies [34, 36] also compared the outcomes between K-wire and screw fixation in lateral condyle fractures in children, but they were excluded from the quantitative analysis because they included patients with nondisplaced and minimally displaced fractures. These studies also reported improved outcomes in the screw-fixation group. A study by Ganeshalingam et al. [36] included 336 patients; 235 patients underwent K-wire fixation, and 101 patients underwent screw fixation. Lower rates of nonunion and superficial infection were reported in the screw fixation group. Cummings et al. [34] included 762 patients from six different institutions; 553 patients were in the K-wire fixation group, and 209 patients were in the screw fixation group. The nonunion rate did not differ between the groups, but superficial infection and elbow stiffness were statistically higher in the K-wire fixation group. Even though they included nondisplaced and minimally displaced fractures, the results correlated with those of our study.

This study has some limitations. First, only four studies were included, leading to a relatively small effect size. Second, the method of reduction and the proportion of types of classification were different, and this may have led to heterogeneity in outcomes. Third, the mean follow-up period in these studies were relatively short. Fourth, only articles published in English was included. Fifth, cost difference between the treatments, including removal of screws after screw fixation, were not identified due to lack of information from the articles, which might be one of the factors that could affect the decision on which fixation method to choose. Lastly, most of the studies included in the quantitative synthesis were retrospective studies. Further prospective studies with a long follow-up period and a large number of patients are warranted.

## Conclusions

The use of screws for fixation after reduction in the treatment of lateral condyle fracture of the humerus in children decreases the risk of superficial infection and elbow stiffness compared with the use of K-wire. Although removal of the implant under general anesthesia is necessary, screw fixation can be considered for better outcomes in displaced lateral condyle fractures of the humerus in children.

### Supplementary Information

**Additional file 1: Supplementary Figure 1**.

**Additional file 2: Supplementary table I**. Search terms for each search engine.

**Additional file 3: Supplementary table II**. 2012 Li [20]. 2016 Gilbert [19]. 2017 Stein [22]. 2019 Thapa [21]. 

## Data Availability

All data generated or analyzed during this study are included in this published article and its supplementary information files.

## Abbreviations

PRISMA: Preferred Reporting Items for Systematic Reviews and Meta-Analyses
MINORS: Methodological Index for Non-Randomized Studies

## References

[1] Okubo H, Nakasone M, Kinjo M, Onaka K, Futenma C, Kanaya F (2019). Epidemiology of paediatric elbow fractures: a retrospective multi-centre study of 488 fractures. J Child Orthop.

[2] Aibara N, Takagi T, Seki A (2022). Late displacement after lateral condylar fractures of the humerus. J Shoulder Elbow Surg.

[3] Bast SC, Hoffer MM, Aval S (1998). Nonoperative treatment for minimally and nondisplaced lateral humeral condyle fractures in children. J Pediatr Orthop.

[4] Greenhill DA, Funk S, Elliott M, Jo CH, Ramo BA (2019). Minimally Displaced Humeral Lateral Condyle Fractures: Immobilize or Operate When Stability Is Unclear?. Journal of Pediatric Orthopaedics.

[5] Kraft DB, Moore TJ, Pargas C, Rogers K, Thacker MM (2023). Minimally Displaced Lateral Humeral Condyle Fractures: Optimizing Follow-up and Minimizing Cost. Journal of Pediatric Orthopaedics.

[6] Launay F, Leet AI, Jacopin S, Jouve JL, Bollini G, Sponseller PD (2004). Lateral humeral condyle fractures in children: a comparison of two approaches to treatment. J Pediatr Orthop.

[7] Pirker ME, Weinberg AM, Höllwarth ME, Haberlik A (2005). Subsequent displacement of initially nondisplaced and minimally displaced fractures of the lateral humeral condyle in children. Journal of Trauma - Injury, Infection and Critical Care.

[8] Flynn JC (1989). Nonunion of slightly displaced fractures of the lateral humeral condyle in children: An update. Journal of Pediatric Orthopaedics.

[9] Silva M, Cooper SD (2015). Closed Reduction and Percutaneous Pinning of Displaced Pediatric Lateral Condyle Fractures of the Humerus: A Cohort Study. Journal of Pediatric Orthopaedics.

[10] Song KS, Kang CH, Min BW, Bae KC, Cho CH, Lee JH (2008). Closed reduction and internal fixation of displaced unstable lateral condylar fractures of the humerus in children. J Bone Joint Surg Am.

[11] Song KS, Shin YW, Oh CW, Bae KC, Cho CH (2010). Closed reduction and internal fixation of completely displaced and rotated lateral condyle fractures of the humerus in children. J Orthop Trauma.

[12] Xie LW, Tan G, Deng ZQ, Liu X, Zhou Y, Zhang H (2022). Impacts of fracture types on success rate of closed reduction and percutaneous pinning in pediatric lateral condyle humerus fractures displaced > 4 mm. Journal of Pediatric Orthopaedics.

[13] Xie LW, Wang J, Deng ZQ, Zhao RH, Chen W, Kang C (2020). Treatment of pediatric lateral condylar humerus fractures with closed reduction and percutaneous pinning. BMC Musculoskelet Disord..

[14] Baharuddin M, Sharaf I (2001). Screw osteosynthesis in the treatment of fracture lateral humeral condyle in children. Med J Malaysia..

[15] Hasler CC, Von Laer L (2001). Prevention of growth disturbances after fractures of the lateral humeral condyle in children. J Pediatr Orthop B.

[16] Loke WP, Shukur MH, Yeap JK (2006). Screw osteosynthesis of displaced lateral humeral condyle fractures in children: a mid-term review. Med J Malaysia..

[17] Sinha S, Kumar A, Meena S, Jameel J, Qureshi OA, Kumar S (2023). K Wires or Cannulated Screws for Fixation of Lateral Condyle Fractures in Children: A Systematic Review of Comparative Studies. Indian J Orthop.

[18] Hardacre JA, Nahigian SH, Froimson AI, Brown JE (1971). Fractures of the lateral condyle of the humerus in children. J Bone Joint Surg Am.

[19] Gilbert SR, MacLennan PA, Schlitz RS, Estes AR (2016). Screw versus pin fixation with open reduction of pediatric lateral condyle fractures. J Pediatr Orthop B.

[20] Li WC, Xu RJ (2012). Comparison of Kirschner wires and AO cannulated screw internal fixation for displaced lateral humeral condyle fracture in children. Int Orthop.

[21] Thapa P, Sapkota K, Wahegaonkar K, Ranjeet N, Onta PR, Thapa UJ (2019). Comparison of Kirschner wires and Cannulated screw internal fixation for displaced lateral humeral condyle fracture in children. Asian J Med Sci..

[22] Stein BE, Ramji AF, Hassanzadeh H, Wohlgemut JM, Ain MC, Sponseller PD (2017). Cannulated lag screw fixation of displaced lateral humeral condyle fractures is associated with lower rates of open reduction and infection than pin fixation. Journal of Pediatric Orthopaedics.

[23] Sterne JAC, Savovic J, Page MJ, Elbers RG, Blencowe NS, Boutron I (2019). RoB 2: a revised tool for assessing risk of bias in randomised trials. BMJ.

[24] Slim K, Nini E, Forestier D, Kwiatkowski F, Panis Y, Chipponi J (2003). Methodological index for non-randomized studies (minors): development and validation of a new instrument. ANZ J Surg.

[25] Mintzer CM, Waters PM, Brown DJ, Kasser JR (1994). Percutaneous pinning in the treatment of displaced lateral condyle fractures. Journal of Pediatric Orthopaedics.

[26] Thomas DP, Howard AW, Cole WG, Hedden DM (2001). Three weeks of Kirschner wire fixation for displaced lateral condylar fractures of the humerus in children. Journal of Pediatric Orthopaedics.

[27] Hope PG, Williamson DM, Coates CJ, Cole WG (1991). Biodegradable pin fixation of elbow fractures in children. A randomised trial. J Bone Joint Surg Br..

[28] Li J, Rai S, Gao Y, Ze R, Tang X, Liu R (2020). Biodegradable pins for lateral condylar fracture of the humerus with an early delayed presentation in children: a retrospective study of biodegradable pin vs. Kirschner wire. BMC Musculoskelet Disord..

[29] Li J, Rai S, Liu Y, Ze R, Tang X, Liu R (2020). Is biodegradable pin a good choice for lateral condylar fracture of humerus in children: A comparative study of biodegradable pin and Kirschner wire. Medicine (Baltimore).

[30] Makela EA, Bostman O, Kekomaki M, Sodergard J, Vainio J, Tormala P (1992). Biodegradable fixation of distal humeral physeal fractures. Clin Orthop Relat Res.

[31] Wirmer J, Kruppa C, Fitze G (2012). Operative treatment of lateral humeral condyle fractures in children. Eur J Pediatr Surg.

[32] Sharma JC, Arora A, Mathur NC, Gupta SP, Biyani A, Mathur R (1995). Lateral condylar fractures of the humerus in children: Fixation with partially threaded 40-mm AO cancellous screws. J Trauma-Injury, Infection and Critical Care.

[33] Shirley E, Anderson M, Neal K, Mazur J (2015). Screw fixation of lateral condyle fractures: Results of treatment. J Pediatr Orthop.

[34] Cummings JL, Schwabe MT, Rivera AE, Sanders J, Denning JR, Neal K (2023). K-wire Versus Screw Fixation in Displaced Lateral Condyle Fractures of the Humerus in Children: A Multicenter Study of 762 Fractures. J Pediatr Orthop.

[35] Franks D, Shatrov J, Symes M, Little DG, Cheng TL (2018). Cannulated screw versus Kirschner-wire fixation for Milch II lateral condyle fractures in a paediatric sawbone model: a biomechanical comparison. J Child Orthop.

[36] Ganeshalingam R, Donnan A, Evans O, Hoq M, Camp M, Donnan L (2018). Lateral condylar fractures of the humerus in children: does the type of fixation matter?. Bone Joint J..

[37] Schlitz RS, Schwertz JM, Eberhardt AW, Gilbert SR (2015). Biomechanical Analysis of Screws Versus K-Wires for Lateral Humeral Condyle Fractures. J Pediatr Orthop.

[38] Sharma HMR, Wilson N (2006). Lateral humeral condyle fractures in children: a comparative cohort study on Screws versus K-wires. J Bone Joint Surg Br.

