# Supplementary figures and images for: K-wire versus screws in the fixation of lateral condyle fracture of humerus in pediatrics: a systematic review and meta-analysis
Source: BMC Musculoskelet Disord. 2023 Aug 12;24:649. doi: 10.1186/s12891-023-06780-5 (PMC10423410; doi:10.1186/s12891-023-06780-5)

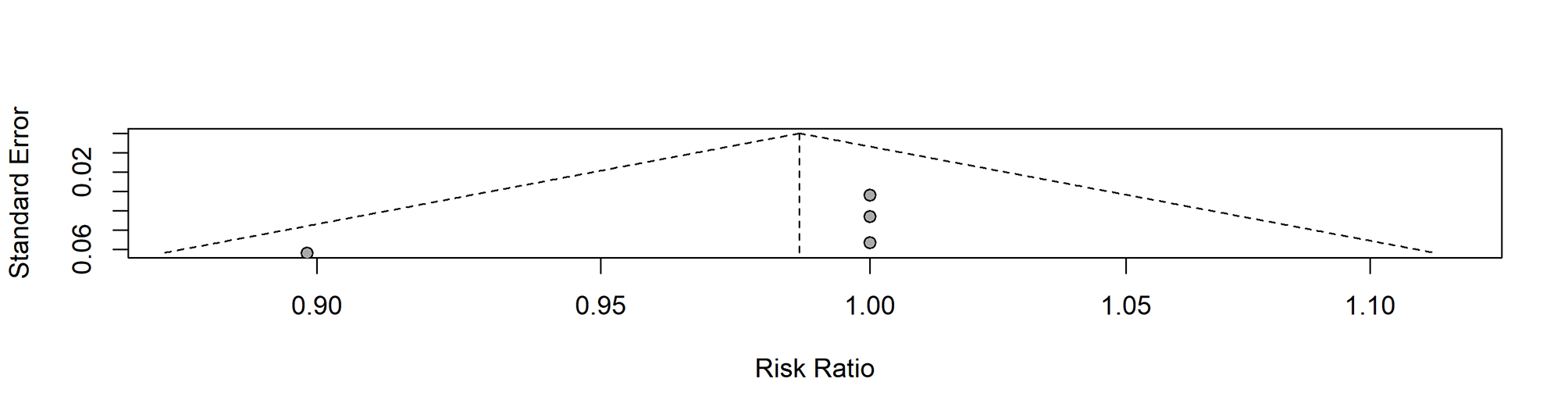

Supplement: Supplementary file 1 — Additional file 1: Supplementary Figure 1. [file 12891_2023_6780_MOESM1_ESM.tif]
