# Supplementary material for: K-wire versus screws in the fixation of lateral condyle fracture of humerus in pediatrics: a systematic review and meta-analysis
Source: BMC Musculoskelet Disord. 2023 Aug 12;24:649. doi: 10.1186/s12891-023-06780-5 (PMC10423410; doi:10.1186/s12891-023-06780-5)
Supplement: Supplementary file 2 — Additional file 2: Supplementary table I. Search terms for each search engine. [file 12891_2023_6780_MOESM2_ESM.docx]

| PubMed | (((((Lateral Condylar Fracture of Humerus) OR (Lateral Condylar Fractures of Humerus)) OR (Lateral Condyle Fracture of Humerus)) OR (Lateral Condyle Fractures of Humerus)) OR (Lateral Humeral Condyle Fractures)) AND ((Peditric) OR (pediatrics) OR (children) OR (child)) |
| --- | --- |
| Embase | ('lateral condylar fracture of humerus' OR (lateral AND condylar AND ('fracture'/exp OR fracture) AND of AND ('humerus'/exp OR humerus)) OR 'lateral condylar fractures of humerus' OR (lateral AND condylar AND ('fractures'/exp OR fractures) AND of AND ('humerus'/exp OR humerus)) OR 'lateral condyle fracture of humerus' OR (lateral AND ('condyle'/exp OR condyle) AND ('fracture'/exp OR fracture) AND of AND ('humerus'/exp OR humerus)) OR 'lateral condyle fractures of humerus' OR (lateral AND ('condyle'/exp OR condyle) AND ('fractures'/exp OR fractures) AND of AND ('humerus'/exp OR humerus)) OR 'lateral humeral condyle fractures' OR (lateral AND humeral AND ('condyle'/exp OR condyle) AND ('fractures'/exp OR fractures))) AND (peditric OR 'pediatrics'/exp OR pediatrics OR 'children'/exp OR children OR 'child'/exp OR child) |
| Cochrane | (Lateral Condylar Fracture of Humerus):ti,ab,kw OR (Lateral Condylar Fractures of Humerus):ti,ab,kw OR (Lateral Condyle Fracture of Humerus):ti,ab,kw OR (Lateral Condyle Fractures of Humerus):ti,ab,kw OR (Lateral Humeral Condyle Fractures):ti,ab,kw |

**Supplementary table I.** Search terms for each search engine
