# Supplementary material for: K-wire versus screws in the fixation of lateral condyle fracture of humerus in pediatrics: a systematic review and meta-analysis
Source: BMC Musculoskelet Disord. 2023 Aug 12;24:649. doi: 10.1186/s12891-023-06780-5 (PMC10423410; doi:10.1186/s12891-023-06780-5)
Supplement: Supplementary file 3 — Additional file 3: Supplementary table II. 2012 Li [20]. 2016 Gilbert [19]. 2017 Stein [22]. 2019 Thapa [21]. [file 12891_2023_6780_MOESM3_ESM.docx]

**2012 Li [19]**

| Methods | Study design: single-center, 2-group, retrospective study  Duration of the study: January 2000 to December 2008 |
| --- | --- |
| Participants | Place of study: Chinese People’s Liberation Army General Hospital  Number of participants assessed: 62 patients (K-wire: 30, Screw: 32)  Inclusion criteria:  - Displaced lateral condyle fractures  Exclusion criteria: not reported  Age: 6.93 years (range 2 to 14 years)  Sex of participants (male/female): 42/20  Side of injury (right/left): 37/25  Types of fracture: Milch I – 14, Milch II - 48 |
| Interventions | Timing of interventions: not reported  Type of surgical intervention:  - Open reduction and K-wire fixation  - Open reduction and 3.5mm AO cannulated screw fixation  Rehabilitation and co-interventions:  - above-elbow plaster splint used for K-wires for 5-6 weeks → After union, K-wire were removed at an outpatient clinic (POD 6-8 weeks)  - above-elbow brace used for screw fixation for 1-2 weeks → After removal of brace, ROM was started → Screws were removed after 8-12 weeks under general anesthesia |
| Outcomes | Length of follow-up: 39.4 months (range 26 to 95 months)  Loss to follow-up: none  Primary outcomes: (at last follow-up): Hardacre clinical outcome  Secondary outcomes: nonunion, avascular necrosis, epiphysial fusion, infection (skin), lateral condyle overgrowth, abnormal carrying angle, limitation of range of motion (loss of extension more than 10 degrees) |

**2016 Gilbert [18]**

| Methods | Study design: single-center, 2-group, retrospective study  Duration of the study: not reported |
| --- | --- |
| Participants | Place of study: School of Medicine, University of Alabama at Birmingham, Birmingham, Alabama, USA  Number of participants assessed: 84 patients (K-wire: 43, Screw: 41)  Inclusion criteria:  - Acute, displaced lateral condyle fractures (>2mm)  - Undergone open reduction and internal fixation  Exclusion criteria: not reported  Age: 5.6 years  Sex of participants (male/female): 59/25  Side of injury (right/left): not reported  Types of fracture: Jakob type 2 – 26, Jakob type 3 - 58 |
| Interventions | Timing of interventions: not reported  Type of surgical intervention:  - Open reduction and K-wire fixation  - Open reduction and 4.0 or 4.5mm cannulated screw fixation  Rehabilitation and co-interventions: not reported |
| Outcomes | Length of follow-up: 6.8 months  Loss to follow-up: none  Primary outcomes: (at last follow-up): Hardacre clinical outcome, time to union, days in cast, nonunion (not healed at greater than 6 months)  Secondary outcomes: delayed union (healing at 3-6 months), avascular necrosis (fishtail deformity or capitellar irregularity), infection (pin-tract infection or infection requiring surgical debridement), lateral condyle overgrowth, limitation of range of motion (loss of extension more than 10 degrees and flexion less than 120 degrees), wound problem, additional operation besides removal of hardware |

**2017 Stein [21]**

| Methods | Study design: single-center, 2-group, retrospective study  Duration of the study: 1998 to 2012 |
| --- | --- |
| Participants | Place of study: Johns Hopkins University, USA  Number of participants assessed: 48 patients (K-wire: 22, Screw: 26)  Inclusion criteria:  - Substantially displaced lateral condyle fractures (Jakob II or III)  - Undergone open reduction and internal fixation with K-wire or screw  - adequate medical records available  Exclusion criteria: not reported  Age: 5.1 years in K-wire fixation group, 5.9 years in screw fixation group  Sex of participants (male/female): 33/15  Side of injury (right/left): 14/34  Types of fracture: Jakob type 2 – 24, Jakob type 3 - 24 |
| Interventions | Timing of interventions: not reported  Type of surgical intervention:  - Open reduction and K-wire fixation  - Open reduction and 4.5mm cannulated screw fixation  Rehabilitation and co-interventions:  a bivalved long-arm fiberglass cast or posterior splint. At the 4-week postoperative follow-up visit, interval healing was confirmed on radiographs, the cast was removed, and the elbow was checked for full ROM. |
| Outcomes | Length of follow-up: 4.3 months  Loss to follow-up: none  Primary outcomes: (at last follow-up): Hardacre clinical outcome, time to union, days in cast  Secondary outcomes: nonunion, delayed union, malunion, avascular capitellum necrosis, fishtail deformity, infection, lateral condyle overgrowth, cubitus valgus, cubitus varus, epiphysiodesis of the distal humerus, limitation of range of motion (loss of extension more than 15 degrees), Infection (Deep infection and superficial infection) |

**2019 Thapa [20]**

| Methods | Study design: single-center, 2-group, prospective study  Duration of the study: Dec 2015 to Dec 2018 |
| --- | --- |
| Participants | Place of study: Manipal Teaching Hospital, Phulbari, Pokhara-11, Kaski, Nepal  Number of participants assessed: 46 patients (K-wire: 23, Screw: 23)  Inclusion criteria:  - Acute (less than 2 weeks), displaced lateral condyle fractures (>2mm)  - Children up to 14 years of age  Exclusion criteria:  - Open fractures  - Injuries in the same elbow  - Anatomically deformed elbows  Age: 6.57 years (range 2 to 12 years)  Sex of participants (male/female): 34/12  Side of injury (right/left): 24/22  Types of fracture: Milch I – 12, Milch II - 34 |
| Interventions | Timing of interventions: not reported  Type of surgical intervention:  - Open reduction and K-wire (1.8 mm x 2) fixation  - Open reduction and 4.0mm cannulated screw fixation  Rehabilitation and co-interventions:  - Upper elbow plaster splint for 4 weeks after K-wire fixation, removal of sutures after 2 weeks, K-wire removal when fracture union was visible on radiographs, after removal of K-wire, elbow ROM exercise was started  - Upper elbow plaster splint for 2 weeks after screw fixation, elbow ROM exercise started after 2 weeks. After 12 weeks, screw was removed under general anesthesia |
| Outcomes | Length of follow-up: 12.4 months in K-wire fixation group, 13.8 months in screw fixation group  Loss to follow-up: 4  Primary outcomes: (at last follow-up): Hardacre clinical outcome, time to union, time to removal of implant  Secondary outcomes: infection (superficial pin-site infection), lateral condyle overgrowth, deformity, range of motion, nonunion, malunion |
